# A Validated Injury Surveillance and Monitoring Tool for Fast Jet Aircrew: Translating Sports Medicine Paradigms to a Military Population

**DOI:** 10.1186/s40798-022-00484-1

**Published:** 2022-07-16

**Authors:** James Wallace, Peter Osmotherly, Tim Gabbett, Wayne Spratford, Theo Niyonsenga, Phil Newman

**Affiliations:** 1 University of Canberra Research Institute for Sport and Exercise (UCRISE), Bruce, ACT Australia; 2 School of Health Sciences, The University of Newcastle, Callaghan, NSW Australia; 3 Gabbett Performance Solutions, Brisbane, QLD Australia; 4 Centre for Health Research, University of Southern Queensland, Ipswich, QLD Australia; 5 Institute of Health and Wellbeing, Federation University, Ballarat, VIC Australia; 6 University of Canberra Health Research Institute, Faculty of Health, Bruce, ACT Australia

**Keywords:** Aircraft, Back pain, Consensus, Delphi technique, Factor analysis, Flying personnel, Musculoskeletal disorder, Neck pain, Pilot

## Abstract

**Background:**

Military populations, including fast jet aircrew (FJA - aka fighter aircrew/pilots), commonly suffer from musculoskeletal complaints, which reduce performance and operational capability. Valid surveillance tools and agreed recordable injury definitions are lacking. Our objective was to develop and then evaluate the validity of a musculoskeletal complaints surveillance and monitoring tool for FJA.

**Methods:**

A Delphi study with international experts sought consensus on recordable injury definitions and important content for use in a surveillance and monitoring tool for FJA. Using these results and feedback from end-users (FJA), the University of Canberra Fast Jet Aircrew Musculoskeletal Questionnaire (UC-FJAMQ) was developed. Following its use with 306 Royal Australian Air Force (RAAF) FJA over 4 × five-month reporting periods, validity of the UC-FJAMQ was evaluated via multi-level factor analysis (MFA) and compared with routine methods of injury surveillance.

**Results:**

Consensus was achieved for: eight words/descriptors for defining a musculoskeletal complaint; six definitions of recordable injury; and 14 domains important for determining overall severity. The UC-FJAMQ was developed and refined. MFA identified three distinct dimensions within the 11 items used to determine severity: operational capability, symptoms, and care-seeking. MFA further highlighted that symptom severity and seeking medical attention were poor indicators of the impact musculoskeletal complaints have upon operational capability. One hundred and fifty-two episodes of time loss were identified, with the UC-FJAMQ identifying 79% of these, while routine methods identified 49%. Despite modest weekly reporting rates (61%), the UC-FJAMQ outperformed routine surveillance methods.

**Conclusions:**

The UC-FJAMQ was developed to specifically address the complexities of injury surveillance with FJA, which are similar to those noted in other military and sporting populations. The results demonstrated the UC-FJAMQ to be sensitive and valid within a large group of FJA over 4 × five-month reporting periods. Adoption of consistent, sensitive, and valid surveillance methods will strengthen the FJA injury prevention literature, ultimately enhancing their health, performance, and operational capability.

**Supplementary Information:**

The online version contains supplementary material available at 10.1186/s40798-022-00484-1.

## Key points

- Injury surveillance among military populations, including FJA, is complex, with valid surveillance tools and agreed recordable injury definitions lacking.
- After seeking and reaching consensus, a tool (UC-FJAMQ) was developed to specifically address the complexities of injury surveillance within military populations including FJA. Evaluation of its validity via multi-level factor analysis and comparison to routine methods of injury surveillance determined it was sensitive and valid.
- Consideration should be given to recordable injury definitions and methods of injury surveillance, as this is paramount in strengthening the military injury prevention literature and ultimately operational capability.

## Introduction

Fast jet aircrew (FJA, also referred to as fighter aircrew, fighter pilots, or high-performance jet aircraft aircrew) operate in a demanding environment [1]. Unsurprisingly, they experience a high prevalence of musculoskeletal complaints (an umbrella term which encompasses concepts including pain and injury), particularly of the neck and back [2]. Such musculoskeletal complaints have been shown to impact operational capability via reduced performance in the jet [2–4], periods of being unable to fly [4–6], dropout during training and early career termination [6].

Before effective and efficient prevention interventions can be developed and implemented, prevention models require the extent of the problem to be first established, followed by identification of aetiological factors [7–9], each of which depends upon high-quality longitudinal data where definitions are consistent. Accurately capturing the burden of musculoskeletal complaints among military personnel, including FJA, has traditionally been difficult due to: access, security, ability to follow-up, individual reluctance to provide medical data, inconsistent medical attention seeking behaviour, regular movements across locations (nationally and internationally), and the resultant inability for consistent follow-up with health care providers [1, 10, 11]. Furthermore, comparing results across studies among military personnel [12, 13] including FJA [1] has been further hampered by heterogeneity of both surveillance methods and ‘recordable injury’ definitions used.

A related and well-researched area of epidemiology, sports injury, has typically used three definitions of a ‘recordable injury’: ‘all complaints’; ‘medical attention’ (where medical attention is received); and ‘time loss’ (where training and/or competition is missed) [14–16]. Each have strengths and limitations including: breadth of injuries likely captured; reproducibility of results; scope of application; and effort required by recorders/observers. It has therefore been highlighted that “one size does not fit all” [15] and thus advocated that surveillance methods should use multiple definitions [15, 17, 18]. Further recommendations for improving validity and sensitivity of surveillance methods include: ensuring measurements are prospective and collected at regular intervals; capturing prevalence as opposed to incidence alone; quantifying severity of functional limitation; being sensitive to early clinical symptoms; and where possible, having athletes themselves record their physical complaints [15, 18, 19].

The Oslo Sports Trauma Research Centre Overuse Injury Questionnaire (OSTRC-O) is a validated and widely used self-reported sports injury surveillance tool that considers such complexities [20]. A similar surveillance tool would be of value with FJA given the similarities they share with athletes, including: continuing to train/fly despite symptoms and degraded performance; varied ability to modify their training/flying schedules; differing levels of access and use of medical practitioners; postponing rest or treatment until off-season/non-flying periods; and experience of both sudden and gradual onset musculoskeletal complaints [4, 21, 22]. Such a tool would be useful in epidemiological studies and when evaluating prevention strategies. It may facilitate early identification of musculoskeletal complaints in FJA settings which have dedicated health provider teams [18], and importantly, it could provide regular data to commanders as to the impact of musculoskeletal complaints upon operational capability.

To date, no consensus has been reached as to which ‘recordable injury’ definition/s should be used in FJA research, nor does a standardised method exist to capture musculoskeletal complaints among FJA. The aims of this study were therefore to:

- Reach consensus on ‘recordable injury’ definitions for use in FJA research;
- Develop a self-reported musculoskeletal complaints surveillance and monitoring tool that: is specific to FJA, yet broad enough it can be used across fast jet airframes, sortie types, and aircrew equipment available; captures prevalence and quantifies severity; and is in accordance with previous recommendations [15, 18, 19];
- Determine the validity of the tool when compared to other surveillance records; and
- Examine psychometric properties of the tool which may aid further refinements.

## Methods

Phase 1 of this study was approved by the University of Canberra Human Research Ethics Panel (UC-HREP, reference-20180145) with cross-institutional approval from the Joint Health Command Low Risk Ethics Panel (JHC-LREP, reference-20180517), while phases two and three were approved by the JHC-LREP (reference-17-018) with cross-institutional approval from the UC-HREP (reference-20170089) as part of a larger project.

### Phase 1—Delphi Study

Phase 1 sought consensus on important content and definitions for use in a musculoskeletal complaints surveillance and monitoring tool for FJA. A Delphi design was used, which is a structured process that seeks to obtain consensus from a group of experts (participants) using a series of questionnaires (rounds) that feeds back responses anonymously from the previous round [23, 24]. The Delphi ran from May to July 2018.

Experts invited to participate were defined as: a) primary authors of peer-reviewed research published in the previous 5 years on musculoskeletal complaints amongst FJA; or b) current members of the North Atlantic Treaty Organization (NATO) aircrew musculoskeletal working group and involved with definitions/questionnaire development; or c) those identified by a member of group b above as they were considered to have expertise in this area. Identified experts were sent an email invitation which gave a project overview including the background, purpose, and participation requirements including informed consent.

Consenting participants were asked to partake in three rounds of online questionnaires (Survey Monkey, SurveyMonkey Inc., California). The questionnaires were informed by previously published work, including: the heterogeneity of injury definitions and surveillance methods used in the FJA literature [1]; the complexities of conducting surveillance with FJA [1]; the similarities of such complexities with athletes [4, 21, 22]; and the recommendations put forward [15, 18, 19] and tools developed [20, 25] in the sports injury literature to address such complexities. A mix of open responses, Likert scales, and ranking responses were used. The questionnaires were developed by researchers JW and PO and piloted by researchers PN, TG, and WS with amendments made following their feedback. Participants had 14 days to respond to each round, with email reminders sent to non-respondents at 12 and 16 days to maximise response rates [26]. A further 7 days between rounds allowed researchers to synthesise results and formulate the subsequent round’s questionnaire.

In round one, participants were asked to provide a mix of open responses, and Likert scales to indicate their level of agreement (LOA) as per Table 1. Open responses were de-identified and qualitatively analysed by two researchers (JW and PO) using content analysis [27]. Both researchers independently identified and grouped similarly themed responses into items and then compared results and discussed any differences until consensus was reached (in the instance where consensus was unable to be reached, input from a third researcher (PN) was to be sought; however, this was not required as consensus for all items was reached between JW and PO). A final list of items was included in subsequent rounds where LOA was sought from participants. For consensus to be achieved, > 75% of participants had to agree or strongly agree, or > 75% had to disagree or strongly disagree.

**Table 1:** Structure of the Delphi study seeking expert group consensus

Round	Input sought from participants
1	**Provide open responses for**
	Words/descriptions that are important when describing to FJA what is a musculoskeletal complaint
	Domains that are important in determining overall severity of a musculoskeletal complaint among FJA
	Body regions that should be selectable/included
	Definitions of recordable injury that should be included beyond medical attention and time loss
	Preference for frequency of completion of the tool by FJA
	**Indicate level of agreement** ^a^** regarding**
	Use of a body chart denoting each area selectable in regards to location of musculoskeletal issue
	Inclusion of definitions of recordable injury (medical attention and time loss)
	Capture of mode of onset (gradual or sudden)
	Capture of status (i.e. new, ongoing, or recurrent)
2 and 3	**Indicate level of agreement **^a^ **regarding items generated from round 1**
	Words/descriptions that are important when describing to FJA what is a musculoskeletal complaint
	Domains that are important in determining overall severity of a musculoskeletal complaint among FJA
	Body regions that should be selectable/included
	Definitions of recordable injury that should be included
	Use of a body chart denoting each area selectable in regard to location of musculoskeletal issue
	Capture of mode of onset (gradual or sudden)
	Capture of status (i.e. new, ongoing, or recurrent)
	**Rank in order of importance items generated from round 1**
	Words/descriptions that are important when describing to FJA what is a musculoskeletal complaint
	Domains that are important in determining overall severity of a musculoskeletal complaint among FJA
	**Provide open responses for**
	Domains (listed as important for determining overall severity) that overlap and thus should be combined
	Preference for frequency of completion of the tool by FJA

*FJA* Fast jet aircrew

^a^ Levels of agreement captured using five-point Likert scale (strongly agree, agree, undecided, disagree, strongly disagree)

For rounds two and three, participants were asked to provide a mix of open responses, indications of LOA, and ranking responses as per Table 1. A summary of group responses from the previous round was provided, and opportunity was provided for participants to further revise their judgements in an effort to reach consensus.

### Phase 2—Development of the Tool and Refinement with Aircrew

A musculoskeletal complaints surveillance and monitoring tool was formulated using results from phase 1. This was circulated within the research team until a draft was agreed upon. Ten current Royal Australian Air Force (RAAF) FJA (of varying experience, rank, and geographical location) provided feedback on the draft (including: comprehension, wording/acceptability, suggestions). Following feedback, the final tool was developed—the University of Canberra Fast Jet Aircrew Musculoskeletal Questionnaire (UC-FJAMQ).

### Phase 3—Evaluation of Psychometric Properties and Comparison with Other Methods

Aircrew enrolled as part of larger observational study where informed consent was provided to allow researchers to analyse the data that were routinely collected from Royal Australian Air Force (RAAF) FJA. A convenience sample of 306 RAAF FJA (mean age 32.9, SD 8.0), including 15 females, from five different airframes (BAE Hawk, F/A-18, F/A-18F, EA/18-G, and F-35) were asked to complete the UC-FJAMQ on a weekly basis using Smartabase (FusionSport, QLD, Australia) online software and smartphone app during four consecutive five-month reporting periods which aligned to flying and training schedules. Where possible, aircrew were sent a reminder email each week and push notifications via the smartphone app to encourage completion.

To establish construct validity of the UC-FJAMQ, exploratory factor analysis was undertaken on the 11 items contributing to the severity score. Intraclass correlation (ICC) was computed for each item to evaluate whether multi-level factor analysis (MFA) was required to ensure that within-individual and between-individual variances were accounted for [28] using MPlus 8.6 [29]. If ICCs were greater than 0.05, MFA was deemed to be required [30]. MFA was undertaken whereby the total correlation matrix was partitioned into within-individual and between-individual components and each submitted to exploratory factor analysis using principal component analysis using SPSS V26 (IBM Corporation, NY, USA). To further verify that MFA was suitable for each partition, the Keiser–Meyer–Olkin measure of sampling adequacy was assessed to ensure a value of 0.6 or greater, and that Bartlett’s test of sphericity was significant with a p value smaller than 0.05 [31]. To determine the number of factors to retain, three methods were simultaneously used to identify: the number of factors with eigenvalues exceeding 1.0, the number of factors remaining above a straight line drawn through the smaller eigenvalues on a scree plot, and the number of factors in the component matrix with multiple loadings above 0.4 [31, 32]. To aid the interpretation of the retained factors, the Oblimin rotation with Keiser normalisation was performed as well [31, 32].

To better understand face validity of the UC-FJAMQ, episodes of time loss from flying were compared between those captured by the UC-FJAMQ and those captured by the routine methods of injury registration for RAAF FJA. At the time of this study, RAAF FJA units had dedicated physiotherapists who worked outside of the normal on-base health centres and within the squadrons to provide FJA with early access to care. In addition to their routine clinical documentation (within the Defence e-health data and information system), they would update a routine injury register which captured information such as date presented, occasions of service, body region/location affected, subsequent injury classification coding [33], whether time loss from flying occurred (defined as: ‘days where scheduled flights were unable to be flown due to their complaint, and subsequent days they were not available to be included on the programme due to their complaint’), and duration of any such time loss. Furthermore, these physiotherapists were tasked with verifying with aircrew any time loss entries made in the UC-FJAMQ to confirm that such entries were valid.

Weekly reporting rates of the UC-FJAMQ took into account the posting movements of aircrew in/out of fast jet units throughout each five-month reporting period so that only the period of time they were posted (i.e. present) to a relevant unit was considered in calculating reporting rates. Those present for an entire reporting period were deemed to have a full-time equivalence (FTE) of 1.0, and those present for only part of a reporting period had a FTE value representative of the proportion of time they were present (i.e. < 1.0).

## Results

### Phase 1—Delphi Study

A total of 18 experts were identified and invited to participate. Of these, 10 partook in the first and subsequent two rounds, representing a 56% response rate, with two participants not completing all three rounds giving a total of nine participants per round. Further detail regarding Delphi participant characteristics is provided in Additional file 1a.

For words/descriptions deemed important for describing what constitutes a musculoskeletal complaint, participants provided 58 responses in round one which were grouped into eight similarly themed items (Table 2). Consensus was reached that all were important, with final rankings of importance from round three provided in Table 2.

**Table 2:** Items generated through the Delphi process and subsequent levels of agreement

Themed items generated from Round 1	Level of agreement achieved in each round ^a^			ROI ^b^
	Round 1	Round 2	Round 3	
**Words/description of musculoskeletal complaint**				
Pain	–	100% A^c^	*–*	1
Discomfort	*–*	100% A^c^	*–*	2
Reduced ROM specifically: enough to limit operational performance	*–*	89% A^c^, 11% U	*–*	3
Stiffness	*–*	89% A^c^, 11% U	*–*	4
Reduced ROM	*–*	100% A^c^	*–*	5
Ache	*–*	56% A, 22% U, 22% D	89% A^c^, 11% D	6
Tingling or numbness	*–*	89% A^c^, 11% U	*–*	7
Tingling or numbness specifically: in fingers	*–*	56% A, 22% U, 22% D	89% A^c^, 11% D	8
**Domains to determine musculoskeletal complaint overall severity**				
Severity of pain	*–*	100% A^c^	*–*	1
Duration of pain	*–*	100% A^c^	*–*	2
Impact on flying performance	*–*	100% A^c^		3
Severity of symptoms	*–*	100% A^c^	*–*	4
Prolonged pain after flying	*–*	88% A^c^, 12% U	*–*	5
Impact on planned flying schedule (including reduced duration, number, and/or intensity of sorties able to be flown)	*–*	100% A^c^	*–*	6
Impact on ability to withstand the Gz required for optimal performance of sorties flown	*–*	67% A, 11% U, 22% D	100% A^c^	7
Impact on concentration while flying	*–*	89% A^c^, 11% U	*–*	8
Impact on use of helmet mounted equipment (e.g. JHMCS or NVG)	*–*	89% A^c^, 11% U	*–*	9
Presence of pain at rest	*–*	67% A, 22% U, 11% D	100% A^c^	10
Impact on sleep	*–*	78% A^c^, 11%U, 11% D	*–*	11
Loss of work days	*–*	67% A, 22% U, 11% D	89% A^c^, 11% D	12
Impact on non-work-related activity	*–*	67% A, 11% U, 22% D	100% A^c^	13
Use of therapeutic intervention (such as input from health care provider, exercise, or medication)	*–*	78% A^c^, 22% D	*–*	14
Presence of psychological stress	*–*	22% A, 44% U, 33% D	44% A, 56% D	15
**Definitions of recordable injury to be included**				
Time loss has occurred (i.e. whether they have been unable to partake in their flying duties as a result of their complaint)	100% A^c^	*–*	*–*	*–*
Attention has been sought from a qualified medical practitioner (e.g. Doctor, Physiotherapist, Osteopath, Chiropractor)	100% A^c^	*–*	*–*	*–*
Contact with a physical training instructor	*–*	22% A, 44% U, 33% D	22% A, 78% D^c^	*–*
Limitation of activities of daily living	*–*	78% A^c^, 22% U	*–*	*–*
Impact on rest/sleep	*–*	67% A, 33% U	100% A^c^	*–*
Limited spinal range of movement	*–*	66% A, 22% U, 11% D	89% A^c^, 11% D	*–*
Use of medication for pain	*–*	78% A^c^, 22% U	*–*	*–*
**Body regions to be listed/selectable**				
Neck or cervical spine	*–*	100% A^c^	*–*	*–*
Upper back or thoracic spine	*–*	100% A^c^	*–*	*–*
Lower back or lumbar spine	*–*	100% A^c^	*–*	*–*
Buttocks	*–*	44% A, 11%U, 44%D	56% A, 44% D	*–*
Shoulder (including the scapula)	*–*	100% A^c^	*–*	*–*
Upper limb	*–*	89% A^c^, 11% U`	*–*	*–*
Lower limb	*–*	67% A, 11% U, 22% D	100% A^c^	*–*
Hip	*–*	33% A, 44% U, 22% D	56% A, 44% D	*–*
Buttocks should be incorporated with lower back/lumbar spine	*–*	44% A, 11% U, 44% D	56% A, 44% D	*–*
Divide neck/cervical spine into upper and lower	*–*	33% A, 11% U, 56% D	11% A, 89% D^c^	*–*
**Other**				
Use of a body chart denoting areas selectable regarding location of musculoskeletal complaint	100% A^c^	*–*	*–*	*–*
Capture of mode of onset (gradual or sudden)	89% A^c^, 11% U	*–*	*–*	*–*
Capture of status (new, ongoing, recurrent)	89% A^c^, 11% U	*–*	*–*	*–*

ROI, rank of importance; ROM, range of motion; JHMCS, joint helmet mounted cueing system; –, not applicable; NVG, night vision goggles; A, agree/strongly agree; D, disagree/strongly disagree; U, undecided

^a^ Percentage indicates proportion of participants per providing a given response; ^b^ Rank of importance is based upon results of round 3; ^c^, Items achieving the predetermined > 75% participant agreement indicating consensus was achieved

For domains deemed important for determining musculoskeletal complaint overall severity, participants provided 60 responses in round one which were grouped into 15 similarly themed items (Table 2). Consensus was reached that 14 were important, with final rankings of importance from round three provided in Table 2. Six suggestions were provided by participants where they felt items overlapped so much that they should be combined (Additional file 1b).

For body regions considered important to be selectable/included, participants provided 54 responses in round one which were grouped into eight regions. Consensus was reached that six were important (Table 2), highlighting that regions beyond the spine should be incorporated. Based upon responses from round one, participants were also asked: should the neck be divided into upper and lower regions (consensus reached was they should not); and should the lumbar area be combined with the buttocks given that multiple definitions define low back pain as localised between the lower costal margin and the gluteal folds [34, 35] (consensus could not be reached) (Table 2). Consensus was reached that a body chart outlining each area selectable should be included.

For ‘recordable injury’ definitions, consensus was reached that time loss from flying duties, and medical attention should be included. Participants put forward five further suggestions, with consensus reached that four should, and one should not, be included (Table 2). Mode of onset (gradual or sudden) and status (new, ongoing, recurrent) both reached consensus they should be included (Table 2). The frequency with which FJA should complete the tool did not reach consensus, with responses ranging from weekly to yearly.

### Phase 2—Development of Tool and Refinement with Aircrew

A draft questionnaire (tool) was developed and agreed upon by the researchers, which consisted of three parts. Part one asked aircrew whether they had experienced a musculoskeletal complaint (‘*a musculoskeletal complaint refers to pain, ache, discomfort, stiffness, or tingling or numbness which may be experienced anywhere in your body’*) in the previous week, and for the location of symptoms marked on a body chart (developed by Smartabase, FusionSport) divided into 18 body regions as used by the OSICS-10.1 [36]. Part two consisted of eight questions regarding severity and duration of their complaint, and the impact upon: flying performance, ability to withstand required g-forces (+ Gz), choice of helmet mounted devices (HMD),^1^ concentration while flying, planned flying schedule, and non-flying-related activities. Part three consisted of seven questions regarding: onset, status, days of flying duties missed (timeloss), medical attention sought, use of symptom-relieving medications, and loss of movement.

Surveillance frequency was set at weekly to minimise recall error and provide more opportunity for early identification/intervention when used as a monitoring tool and was consistent with similar validated tools [20, 25].

Using the Delphi findings, a severity score was formulated based upon 11 items including: symptom severity, symptom duration, impact upon required + Gz, impact upon flying performance, influence on HMD use, impact on concentration while flying, impact on planned flying schedule, impact on non-flying-related activities, days of flying duties missed, medical attention sought, and any use of medications. Each question contributed 10 points to an overall severity score out of 110 (0 indicating no complaint and 110 indicating the most severe).

Feedback from aircrew indicated that the questionnaire was easy to understand, and the reasoning for each question was apparent. There was consistent appreciation for in-built logic whereby no further questions were imposed if they answered ‘no’ regarding had they experienced a musculoskeletal complaint. Two aircrew indicated combining/reducing questions would be ideal but were unsure which to remove/combine. It was suggested the medical system should capture questions relating to movement loss and seeking medical care. For the medication question it was suggested there should be the ability to select medication type to increase openness among aircrew to overcome any perceived risk of having the medical system unnecessarily ground them if it is not apparent that the medication taken was permitted.

The final questionnaire developed following aircrew feedback (UC-FJAMQ) is provided in Additional file 1c.

### Phase 3—Evaluation of Psychometric Properties

Number of FJA included in each of the four reporting periods is provided in Table 3.

**Table 3:** Data pertaining to Phase 3—comparison between surveillance methods across the four reporting periods

	Reporting period				Total (mean)
	1	2	3	4	
**Number of participants**					
Total participants ^a^	245	252	246	238	306 (245)
Adjusted for FTE ^b^	224.9	233.9	230.5	225.3	(228.7)
**UC-FJAMQ**					
Response rate	50%	61%	70%	64%	(61%)
Total entries	2,593	3,464	3,897	3,542	13,496 (3,374)
Entries indicating a MSK complaint had been experienced ^c^	467	581	550	528	2,126 (532)
**Episodes of time loss from flying**					
Identified by UC-FJAMQ	29	34	20	37	120 (30)
Identified by routine surveillance methods	13	22	16	24	75 (19)
Identified overall ^d^	33	45	29	45	152 (38)

^a^Number of fast jet aircrew enrolled in study and in flying role for that period

^b^Number of aircrew enrolled in study and in flying role for that period, but adjusted for full-time equivalence (FTE)

^c^MSK = musculoskeletal

^d^After removing duplicates between methods

A total of 2127 questionnaires were completed where aircrew (*n* = 215) indicated they had suffered a musculoskeletal complaint in a given week. ICC’s for each of the 11 items (Additional file 1d) reinforced the need to undertake MFA. Correlational matrices for both within-individual and between-individual partitions are provided in Additional file 1d. Kaiser–Meyer–Olkin values were 0.818 and 0.718 for the within-individual and between-individual analyses (respectively) exceeding the recommended value of 0.6 [31], and Bartlett’s test of sphericity for each reaching statistical significance (*p* < 0.001), supporting the factorability of each correlation matrix.

#### Within-Individual Analysis

**Fig. 1:**
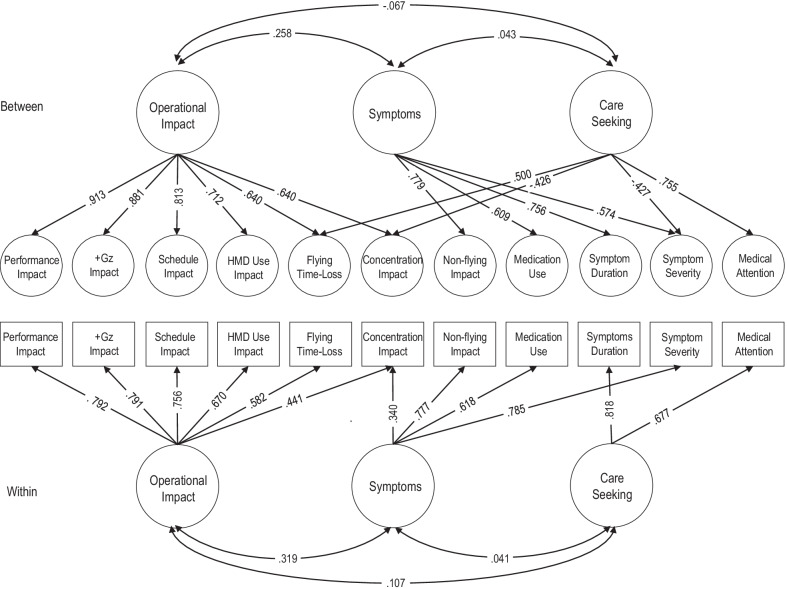
Multi-level factor analysis of UC-FJAMQ. (Factor loadings < 0.3 not shown)

MFA for the within-individual analyses revealed three factors with eigenvalues exceeding 1.0, explaining 34.3%, 11.4%, and 11.1% of the variance, respectively. Scree plot examination reinforced the presence of three factors, as did component matrix examination [32]. Three factors were extracted and rotated using Oblimin with Keiser normalisation. The factor loadings are visually displayed in Fig. 1 and tabulated in Additional file 1e.

As Fig. 1 illustrates, the first factor (*operational impact*) appeared to reflect flying-related performance impact. The second factor (*symptoms*) captured musculoskeletal complaints which had higher symptom severity and greater impact outside of flying and caused aircrew to use medications. The third factor (*care-seeking*) appeared to capture musculoskeletal complaints where medical attention was sought for symptoms that were persistent. Correlations between factors were small to moderate (Fig. 1).

#### Between-Individual Analysis

MFA for the between-individual analyses revealed three factors with eigenvalues exceeding 1.0, explaining 40%, 15.5%, and 11.9% of the variance, respectively. Scree plot examination reinforced the presence of three factors, as did component matrix examination [32]. Three factors were extracted and rotated using Oblimin with Keiser normalisation. The factor loadings are again visually displayed in Fig. 1 and tabulated in Additional file 1e.

Similar factors were identified, with the first (*operational impact*) reflecting flying-related performance impact. The second factor (*symptoms*) appeared to capture musculoskeletal complaints with longer symptom duration, higher symptom severity, greater impact outside of flying, and use of medications to reduce such symptoms and impact. The third factor appeared to capture musculoskeletal complaints where medical attention was sought and time loss incurred, but contrasted with lower symptom severity and concentration impact. Correlations between factors were small to moderate (Fig. 1).

### Phase 3—Comparison with Other Methods

Average weekly response rate for the UC-FJAMQ was 61% for each reporting period (Table 3). Overall, the UC-FJAMQ captured 120 time loss episodes (mean of 30 per reporting period), while routine methods captured 75 time loss episodes (Table 3). After removal of duplicates, 152 time loss episodes were captured overall. Of the time loss episodes captured by routine methods, 57% (43/75) were also captured by the UC-FJAMQ, which increased to 82% when accounting for those who had not been compliant with UC-FJAMQ entries during that episode. The UC-FJAMQ captured 77 time loss episodes not captured by routine methods, indicating the UC-FJAMQ captured 79% (120/152) of all time loss episodes, whereas the routine methods only captured 49% (75/152).

## Discussion

Using a panel of international experts, this study has reached consensus on recordable injury definitions for use in future FJA research. Furthermore, consensus was reached on important content for use in a musculoskeletal complaints surveillance and monitoring tool that addresses the various complexities of surveillance with FJA, which are similar to those noted in other military and sporting populations. A final tool was then developed (UC-FJAMQ) which included feedback from end-users (FJA). Following its use with RAAF FJA over four 5-month reporting periods, analysis demonstrated that it is robust and superior to routine methods of injury surveillance.

Previous surveillance in FJA has predominantly focussed on descriptors such as ‘back pain’ or ‘neck pain’. However, consensus reached in our study indicates that other descriptors such as stiffness, reduced ROM, and tingling or numbness are also important. These align with broader terms used in other surveillance systems [20, 25] and more inclusive contemporary definitions of injury and health problems [14]. The consensus that body regions beyond the spine should be included highlighted the need for broader surveillance. Thus, the UC-FJAMQ can capture the extent that FJA suffer from non-spinal musculoskeletal complaints.

Consensus was reached that six separate definitions of recordable injury were important (Table 2), two of which (time loss and medical attention) were to be expected given their use in sports and military injury research. However, the other four (limitation of activities of daily living, impact on rest/sleep, limited spinal range of motion, use of medication) were not anticipated. This may reflect the field of FJA injury epidemiology being less established than fields such as sports injury where greater time and effort have been invested in creating and refining their definitions, or, that the group of experts in this study have identified important subgroups of FJA suffering from a musculoskeletal complaint. For instance, the large importance in aviation settings placed upon aircrew’s fatigue and medication usage may explain the inclusion of impact upon rest/sleep and use of medication definitions. Similarly, the requirement for FJA to move their necks through large ranges of motion in-flight [22, 38] may also explain the inclusion of the limited spinal range of motion definition. Nonetheless, reaching a consensus on such definitions is important, as it will encourage future research to use definitions that are comparable between studies.

Previous surveillance in FJA has predominantly used pain severity alone to describe severity of musculoskeletal complaints. However, 14 domains were deemed important, thereby allowing functional impact and operational capability to also be considered in an overall severity score that would otherwise not be captured. This not only permits operational impact to be measured, further understood, and compared across future studies, but offers a more accurate method to regularly update commanders of the operational impact of musculoskeletal complaints among their aircrew, particularly when aircrew continue to fly despite being symptomatic and performing sub-optimally.

Results of the MFA revealed three distinct factors/dimensions: operational impact, symptoms, and care-seeking. Across the within-individual and between-individual analyses, the first dimension (operational impact) remained consistent. This dimension offers an important subscale to measure severity of operational impact beyond traditional measures of symptom severity or time loss duration. Interestingly, symptom severity demonstrated minimal relationship with this dimension, as demonstrated by its low loading values in each analysis (Additional file 1e) highlighting operational impact cannot be explained by symptom severity alone. The second (symptoms) and third (care-seeking) dimensions again emerged across the analyses; however, some differences appeared, reflecting the individual nature of symptoms and care-seeking among aircrew. The negative factor loadings (symptom severity, concentration impact) seen in the care-seeking dimension in the between-individual analysis likely reflect the negative correlations between symptom severity and time loss, and the absence of linear relationship between symptom severity and concentration impact in the between-aircrew analysis (Additional file 1d). Again, this reinforces that symptom severity, time loss, concentration impact, and care-seeking behaviours are likely affected by other factors including: variable access to care and individual thresholds for seeking care, and the highly variable demands across flying programmes and sortie types.

The UC-FJAMQ outperformed the routine methods utilised by the embedded physiotherapists, whereby the UC-FJAMQ captured 79% of the net time loss injuries captured, whereas routine methods captured 49%. Given time loss episodes entered into the UC-FJAMQ were cross checked by embedded physiotherapists, such discrepancies are less likely to represent errors made by FJA. Previous work among occupational, sporting, and military populations has highlighted that surveillance systems are affected by under reporting (i.e. musculoskeletal complaints are not captured). Common themes for this include: recordable injury definitions being too narrow [15, 19], reliance upon medical attention being sought (and from the appropriate providers) [15, 39, 40], and fear of negative consequences [39, 41–44]. The self-report OSTRC-O has shown to reduce underreporting by using broader recordable injury definitions, and removing reliance upon medical attention being sought [20]. Given we did not use a broader recordable injury definition (i.e. we compared rates of time loss captured by both methods), the superiority of the UC-FJAMQ may be better explained by: periods of reduced access to physiotherapy care (e.g. FJA away on exercise without physiotherapists; physiotherapist absences; non-flying duties were prioritised; or symptoms were of short duration and thus resolved prior to access); FJA perceived embedded physiotherapy care to offer little benefit [5, 42]; FJA perceived the self-report nature of the UC-FJAMQ having less potential for negative consequences compared to seeking physiotherapy care; and the complexities of using time loss definitions in FJA settings.

**Fig. 2:**
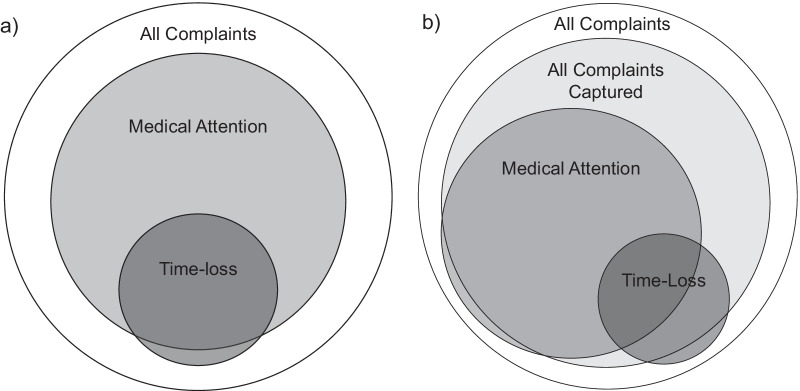
Interactions between various recordable definitions of injury. **a** is based upon that published by Clarsen & Bahr (15), with permission. Where circle size represents the relative number of incidents likely to be registered (not to scale). **b** builds upon this by incorporating the role of surveillance methods, highlighting: i) the overlap of time loss injuries whereby medical attention is not always sought, and time loss injuries are not always captured by surveillance systems; ii) the overlap of medical attention injuries whereby medical encounters are not always captured or occur outside of the provided medical provider resources; and iii) therefore the importance of not relying on medical attention based surveillance systems alone

Figure 2a illustrates the role that the breadth of recordable injury definition has on injuries captured as previously outlined by Clarsen and Bahr [15]. As our findings highlight, it is also important to consider method/s of surveillance. Figure 2b illustrates that reliance upon traditional methods of surveillance, such as those reliant upon medical attention being sought, can under report the true rates of injury even for definitions such as time loss, particularly in settings where time loss can be less definitive (e.g. among FJA, other military personnel, endurance athletes). While it is unknown how many actual musculoskeletal complaints and time loss injuries occurred with RAAF FJA during the reporting periods, our findings place importance upon the use of self-report tools, such as the UC-FJAMQ, to increase the accuracy of injury surveillance. While the UC-FJAMQ was superior to routine methods, it did not capture all of those time loss episodes captured by routine methods, which supports previous advocacy for multisource surveillance systems [40].

## Strengths and Limitations

The strengths of our study are embedded in the conceptual and analysis methodology used. First, we opted for a Delphi study as it: allowed inclusion of a variety of individuals across diverse locations; used anonymity allowing ideas to be presented and reacted to in an unbiased manner; and ensured all participants opinions were weighted equally [23, 45]. Second, we used a longitudinal study design with data collected at multiple time points. Third, with repeated responses to the UC-FJAMQ, the MFA approach was adopted to handle the two sources of variability in the data, within-individual (due to repeated measurements of the same individual over time) and between-individual (due to the sampled aircrew) variations. The approach allowed use of data from all aircrew sampled and all questionnaires completed. Fourth, our study is powered by a large total sample (*n* = 306 aircrew) encompassing multiple airframes and FJA groups (pilots and air combat officers; trainees, instructors and operational aircrew), spanning four 5-month flying periods, with a total of 2127 questionnaires included in the MFA. Lastly, given the strong international participation in the Delphi process, the large encompassing population of RAAF FJA included, and the duration of the study, we are confident the UC-FJAMQ is transferable to other nations FJA populations. However, future studies that translate the UC-FJAMQ to other languages may benefit from some degree of validation.

Our study, however, has some limitations. The first was the number of Delphi participants. This small pool of participants was not surprising given the niche area of FJA injury epidemiology; however, similar sample sizes are not uncommon in Delphi studies [46] given methodological recommendations emphasise participant representativeness and expertise, as opposed to statistical sample size calculations [47]. A second limitation was the moderate response rate of aircrew. Nevertheless, our results demonstrated the UC-FJAMQ still outperformed routine methods of injury surveillance, and did so in a ‘real-world setting’ whereby a large population of FJA used the tool over a long (2-year) period. While others have removed participants who failed to respond every week from their analyses [48], we opted to include such participants in order to accurately reflect the application with FJA. Lastly, we did not provide further detail regarding the epidemiological data collected by the UC-FJAMQ during the reporting periods (such as rates of injury per body region, and reported for each: recordable injury definition, gender, FJA role, airframe, etc.) as this was beyond the scope of the current study.

## Conclusions

This study has reached consensus on recordable injury definitions for use with FJA. Further consensus from international experts, and feedback from FJA, facilitated the development of a FJA-specific musculoskeletal complaints surveillance and monitoring tool (UC-FJAMQ) which addresses many of the complexities of conducting injury surveillance among military populations. Analysis of its validity following use with RAAF FJA over four 5-month reporting periods demonstrated it is psychometrically robust, and outperformed routine methods of injury surveillance despite moderate reporting rates. Given these findings, researchers and embedded healthcare teams among FJA should strongly consider the use of the UC-FJAMQ in addition to the definitions of recordable injury that reached consensus. Consistent use of definitions combined with more accurate surveillance tools will allow for greater rigour, easier comparison across future studies, and increased ability for early identification of musculoskeletal complaints. Ultimately, this will lead to enhanced health, performance, and operational capability of FJA.

## Supplementary Information

**Additional file 1:** Delphi participant characteristics; Domains used to determine severity that participants felt overlapped so much they should be combined; the UC-FJAMQ; Within and between-individuals correlation matrices; and Factor loadings for within- and between-individuals analyses.

## Data Availability

The data sets used for analysis in this study may be provided upon reasonable request pending approval for release from relevant organisations within Australian Defence Organisation.

## Abbreviations

+ Gz: Gravitational forces in the + *z* direction
FJA: Fast jet aircrew
FTE: Full-time equivalence
HMD: Helmet mounted devices
ICC: Intraclass correlation
JHC-LREP: Joint Health Command Low Risk Ethics Panel
LOA: Level of agreement
MFA: Multi-level factor analysis
NATO: North Atlantic Treaty Organization
OSTRC-O: Oslo Sports Trauma Research Centre Overuse Injury Questionnaire
RAAF: Royal Australian Air Force
UC-FJAMQ: University of Canberra Fast Jet Aircrew Musculoskeletal Questionnaire
UC-HREP: University of Canberra Human Research Ethics Panel

## Footnotes

1 HMDs such as JHMCS and NVG offer FJA improved situational awareness and abilities with cueing weapon systems and sensors. However, such equipment come with increased weight and forward shifted centre of gravity, which place further loads upon the necks of FJA [37]. Unpublished data has shown that it is not uncommon for FJA to forego use of HMDs (and their inherent benefits) in an effort to reduce loads upon their necks as a response to current neck pain, or as a preventative measure.
