# Supplementary material for: A Validated Injury Surveillance and Monitoring Tool for Fast Jet Aircrew: Translating Sports Medicine Paradigms to a Military Population
Source: Sports Med Open. 2022 Jul 16;8:92. doi: 10.1186/s40798-022-00484-1 (PMC9288569; doi:10.1186/s40798-022-00484-1)
Supplement: Supplementary file 1 — Additional file 1: Delphi participant characteristics; Domains used to determine severity that participants felt overlapped so much they should be combined; the UC-FJAMQ; Within and between-individuals correlation matrices; and Factor loadings for within- and between-individuals analyses. [file 40798_2022_484_MOESM1_ESM.docx]

| **Additional Files** |
| --- |

**A validated injury surveillance and monitoring tool for fast jet aircrew: Translating sports medicine paradigms to a military population**

Sports Medicine – Open

James Wallace^1^, Peter Osmotherly^2^, Tim Gabbett^3, 4, 5^, Wayne Spratford^1^, Theo Niyonsenga^5^, Phil Newman^1^.

^1^ University of Canberra Research Institute for Sport and Exercise (UCRISE), Bruce ACT, Australia.

^2^ School of Health Sciences, The University of Newcastle, Callaghan NSW, Australia.

^3^ Gabbett Performance Solutions, Brisbane QLD Australia.

^4^ Centre for Health Research, University of Southern Queensland, Ipswich QLD, Australia.

^5^ Institute of Health and Wellbeing, Federation University, Ballarat VIC, Australia.

^6^ University of Canberra Health Research Institute, Faculty of Health, Bruce ACT, Australia.

Correspondence to: [james.wallace@canberra.edu.au](mailto:james.wallace@canberra.edu.au)

| **Additional file 1a** |
| --- |

| **Table.** Delphi participant characteristics | |
| --- | --- |
| Recruitment source | Total n = 10 |
| Primary author of research published in previous 5 years | 7 |
| Member of recent NATO working group and specifically involved with definitions & questionnaire development | 3 |
| Put forward by NATO working group as having expertise in this area | 0 |
| Country |  |
| Australia | 1 |
| Belgium | 1 |
| Denmark | 1 |
| Finland | 3 |
| Republic of Korea | 1 |
| Poland | 1 |
| United Kingdom | 1 |
| United States of America | 1 |

| **Additional file 1b** |
| --- |

| **Table.** Domains used to determine severity that participants felt overlapped so much they should be combined |
| --- |
| Severity of pain AND severity of symptoms |
| Duration of pain AND prolonged pain after flying |
| Presence of pain at rest AND impact on rest/sleep |
| Impact on flying performance AND impact on planned flying schedule |
| Impact on flying performance AND impact on concentration while flying |
| Impact on planned flying schedule AND impact on ability to withstand the Gz required for optimal performance of sorties flown |

| **Additional file 1c** |
| --- |

| **University of Canberra Fast Jet Aircrew Musculoskeletal Questionnaire (UC-FJAMQ)** |
| --- |

**The term “MSK complaint” refers to pain, ache, discomfort, stiffness, or tingling or numbness which may be experienced anywhere in your body.**

Select the alternative that is most appropriate for you, and in the case that you are unsure, try to give an answer as best as you can anyway.

| **Part One** |
| --- |

1. Have you experienced a MSK complaint during the past week?
   - Yes
   - No

[*** In-built logic: all following questions would only appear if they answer ‘yes’ to the above question]

1. Please select area that best describes the location of this MSK complaint.

If the MSK complaint spans across several locations please select the main area.

If you have multiple MSK complaints please complete a separate registration of each one.

|  | Anatomical sites* selectable by aircrew |
| --- | --- |
| 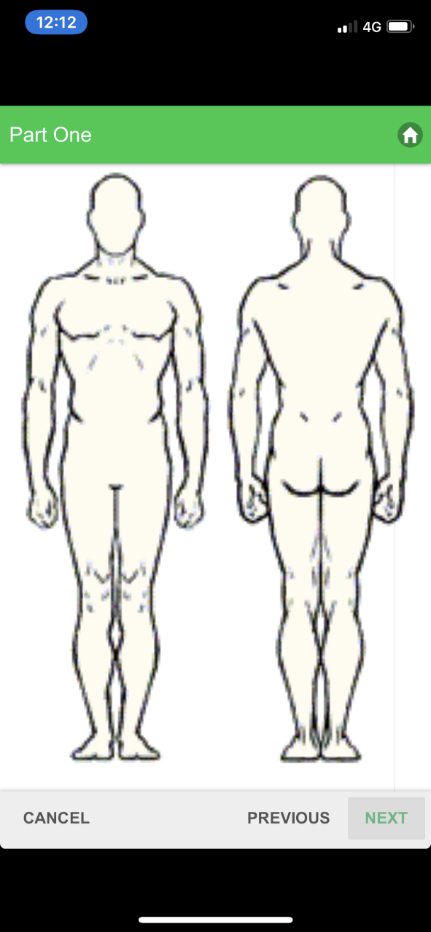 | Head  Neck  Shoulder  Upper arm  Elbow  Forearm  Wrist and hand  Chest  Trunk and abdomen  Thoracic spine  Lumbar spine  Pelvis and buttock  Hip and groin  Thigh  Knee  Lower leg  Ankle  Foot |
|  | **as outlined by OSICS 10-1* |

[***The above body chart is that displayed in the Smartabase (Fusion Sport, Brisbane Australia) version of this questionnaire, and uses shading to highlight the 18 sites as outlined by OSICS 10-1. The table can be displayed as an alternative option for aircrew to select the location of their symptoms].

| **Part Two** |
| --- |

1. How would you describe the severity of pain/symptoms of your MSK complaint during the past week?

|  | | 0 | 1 | 2 | 3 | 4 | 5 | 6 | 7 | 8 | 9 | 10 |  |
| --- | --- | --- | --- | --- | --- | --- | --- | --- | --- | --- | --- | --- | --- |
| Nil | |  |  |  |  |  |  |  |  |  | Severe Pain/ Symptoms | |  |

1. How long did your MSK complaint persist this past week?

|  | □ | □ | □ | □ | □ |  |
| --- | --- | --- | --- | --- | --- | --- |
| < 1 hr | | 1 - 24 hours | 1-3 days | 4-5 days | >5 days | |

1. Has your MSK complaint affected your flying performance during the past week?

(this may include your movements within the cockpit, ability to keep tally, accuracy and timing of tasks, flying the jet to the full capacity required for optimal performance)

|  | | 0 | 1 | 2 | 3 | 4 | 5 | 6 | 7 | 8 | 9 | 10 |  |
| --- | --- | --- | --- | --- | --- | --- | --- | --- | --- | --- | --- | --- | --- |
| No Impact | |  |  |  |  |  |  |  |  |  | Could not fly at all | |  |

1. Has your MSK complaint impacted your ability to withstand the G required for optimal performance of sorties flown during the past week?

|  | | 0 | 1 | 2 | 3 | 4 | 5 | 6 | 7 | 8 | 9 | 10 |  |
| --- | --- | --- | --- | --- | --- | --- | --- | --- | --- | --- | --- | --- | --- |
| No Impact | |  |  |  |  |  |  |  |  |  | Could not fly at all | |  |

1. Has a MSK complaint influenced your choice to use of helmet mounted equipment such as JHMCS or NVG during the past week?

|  | □ |  |  |  | □ | □ | □ | □ |  |
| --- | --- | --- | --- | --- | --- | --- | --- | --- | --- |
| Not  applicable | | |  | Nil  influence | | Some  influence | Unable to use NVG/JHMCS at all | Could not fly  at all | |

1. Has a MSK complaint affected your concentration while flying during the past week?

|  | | 0 | 1 | 2 | 3 | 4 | 5 | 6 | 7 | 8 | 9 | 10 |  |
| --- | --- | --- | --- | --- | --- | --- | --- | --- | --- | --- | --- | --- | --- |
| No Impact | |  |  |  |  |  |  |  |  |  | Could not fly at all | |  |

1. Has a MSK complaint affected your planned flying schedule during the past week?

(this may include changes to the number, duration, and/or intensity of sorties)

|  | | 0 | 1 | 2 | 3 | 4 | 5 | 6 | 7 | 8 | 9 | 10 |  |
| --- | --- | --- | --- | --- | --- | --- | --- | --- | --- | --- | --- | --- | --- |
| No Impact | |  |  |  |  |  |  |  |  |  | Could not fly at all | |  |

1. Has a MSK complaint impacted non flying related activities (including sleep, at rest, and other activities of daily living) during the past week?

|  | | 0 | 1 | 2 | 3 | 4 | 5 | 6 | 7 | 8 | 9 | 10 |  |
| --- | --- | --- | --- | --- | --- | --- | --- | --- | --- | --- | --- | --- | --- |
| No Impact | |  |  |  |  |  |  |  |  |  | Major Impact | |  |

| **Part Three** |
| --- |

1. Please state the number of days over the past week that you have had to completely miss flying duties due to this MSK complaint?

|  | □ | □ | □ | □ | □ | □ | □ | □ |  |
| --- | --- | --- | --- | --- | --- | --- | --- | --- | --- |
| 0 days | | 1 day | 2 days | 3 days | 4 days | 5 days | 6 days | 7 days | |

1. Please indicate if you have seen any of the following regarding this MSK complaint:

- Doctor/AVMO, Physiotherapist, Nurse or Medic, or Other (such as Osteopath or Chiropractor)

□ Yes □ No

1. Do you feel that you have you had any loss of movement as a result of your MSK complaint

□ Yes □ No

1. Have you used any medication to reduce the severity of this MSK complaint

□ Yes □ No

[*** In-built logic: if answered ‘*yes*’, a dropdown box appears asking them to tick one of three options: *Panadol*, *Nurofen*, *Other*]

1. Please indicate if the onset of this MSK complaint was:
   - Sudden onset (you remember a distinct moment it started)
   - Gradual onset (you do NOT remember a distinct moment that it started)
2. Is this the first time that you have experienced this MSK complaint?
   - Yes, this is the first time
   - No, this is an ongoing MSK complaint and reported in the last MSK report
   - No, I have experienced it prior, but has only returned in the last week
3. Have you experienced any other MSK complaints during the past 7 days?
   - Yes [*** In-built logic: If “*yes*”, questionnaire starts again so that another MSK complaint can be detailed]
   - No [*** In-built logic: If “*no*”, the questionnaire finishes]

| **Additional file 1d** |
| --- |

| **Table**. Within and between-individuals correlation matrices | | | | | | | | | | | |
| --- | --- | --- | --- | --- | --- | --- | --- | --- | --- | --- | --- |
|  | Performance Impact | +Gz Impact | Schedule Impact | HMD use Impact | Flying Time-Loss | Concentration Impact | Non-flying Impact | Medication use | Symptom Duration | Symptom Severity | Medical attention |
| **Between-Individuals Matrix** | | | | | | | | | | | |
| Performance Impact | 1.000 |  |  |  |  |  |  |  |  |  |  |
| +Gz Impact | 0.185 | 1.000 |  |  |  |  |  |  |  |  |  |
| Schedule Impact | 0.307 | -0.018 | 1.000 |  |  |  |  |  |  |  |  |
| HMD use Impact | 0.154 | 0.059 | 0.543 | 1.000 |  |  |  |  |  |  |  |
| Flying Time-Loss | 0.294 | 0.046 | 0.752 | 0.334 | 1.000 |  |  |  |  |  |  |
| Concentration Impact | 0.324 | 0.045 | 0.936 | 0.529 | 0.777 | 1.000 |  |  |  |  |  |
| Non-flying Impact | 0.105 | 0.166 | 0.355 | 0.399 | 0.107 | 0.476 | 1.000 |  |  |  |  |
| Medication use | 0.276 | 0.076 | 0.646 | 0.392 | 0.522 | 0.742 | 0.535 | 1.000 |  |  |  |
| Symptom Duration | 0.524 | 0.443 | 0.444 | 0.188 | 0.504 | 0.530 | 0.240 | 0.322 | 1.000 |  |  |
| Symptom Severity | -0.089 | 0.265 | 0.007 | -0.140 | -0.167 | 0.000 | 0.165 | 0.214 | 0.079 | 1.000 |  |
| Medical attention | 0.158 | 0.305 | 0.215 | 0.090 | 0.228 | 0.227 | 0.157 | 0.261 | 0.390 | 0.268 | 1.000 |
| **Within-Individuals Matrix** | | | | | | | | | | | |
| Performance Impact | 1.000 |  |  |  |  |  |  |  |  |  |  |
| +Gz Impact | 0.001 | 1.000 |  |  |  |  |  |  |  |  |  |
| Schedule Impact | 0.333 | 0.048 | 1.000 |  |  |  |  |  |  |  |  |
| HMD use Impact | 0.163 | -0.007 | 0.422 | 1.000 |  |  |  |  |  |  |  |
| Flying Time-Loss | 0.275 | -0.005 | 0.502 | 0.161 | 1.000 |  |  |  |  |  |  |
| Concentration Impact | 0.331 | 0.012 | 0.811 | 0.369 | 0.521 | 1.000 |  |  |  |  |  |
| Non-flying Impact | 0.086 | 0.097 | 0.310 | 0.230 | 0.128 | 0.328 | 1.000 |  |  |  |  |
| Medication use | 0.254 | 0.079 | 0.592 | 0.360 | 0.330 | 0.644 | 0.444 | 1.000 |  |  |  |
| Symptom Duration | 0.480 | 0.161 | 0.311 | 0.121 | 0.261 | 0.350 | 0.198 | 0.326 | 1.000 |  |  |
| Symptom Severity | 0.080 | 0.235 | 0.163 | 0.066 | 0.102 | 0.152 | 0.111 | 0.117 | 0.046 | 1.000 |  |
| Medical attention | 0.294 | 0.079 | 0.276 | 0.131 | 0.210 | 0.270 | 0.146 | 0.243 | 0.322 | 0.074 | 1.000 |
| **Intraclass Correlation (ICC)** | | | | | | | | | | | |
|  | 0.284 | 0.484 | 0.355 | 0.164 | 0.322 | 0.289 | 0.135 | 0.217 | 0.341 | 0.238 | 0.386 |
| HMD: helmet mounted devices | | | | | | | | | | | |

| **Additional file 1e** |
| --- |

| **Table**. Factor loadings for within- and between-individuals analyses | | | | | | | |
| --- | --- | --- | --- | --- | --- | --- | --- |
|  | Between-individuals | | |  | Within-individuals | | |
| Item | 1 | 2 | 3 |  | 1 | 2 | 3 |
| Performance Impact | .913 | .119 | -.114 |  | .792 | .220 | -.063 |
| +Gz Impact | .881 | .067 | -.161 |  | .791 | .197 | -.038 |
| Schedule Impact | .813 | .081 | .228 |  | .756 | .097 | .073 |
| HMD use Impact | .712 | -.147 | -.005 |  | .670 | -.135 | -.072 |
| Flying Time-Loss | .640 | -.022 | .500 |  | .582 | -.131 | .260 |
| Concentration Impact | .640 | .221 | -.426 |  | .441 | .340 | -.144 |
| Non-flying Impact | .222 | .779 | -.217 |  | .012 | .777 | .119 |
| Medication use | .082 | .609 | .230 |  | .026 | .618 | .087 |
| Symptom Duration | -.186 | .756 | .235 |  | -.126 | .132 | .818 |
| Symptom Severity | .113 | .574 | -.427 |  | .010 | .785 | -.083 |
| Medical attention | -.014 | .286 | .755 |  | .133 | -.013 | .677 |
| Note: Underlined figures represent loadings greater than 0.30 | | | | | | | |
